# Supplementary material for: Alterations of Glycosphingolipid Glycans and Chondrogenic Markers during Differentiation of Human Induced Pluripotent Stem Cells into Chondrocytes
Source: Biomolecules. 2020 Dec 1;10(12):1622. doi: 10.3390/biom10121622 (PMC7760376; doi:10.3390/biom10121622)
Supplement: Supplementary file 1 [file biomolecules-10-01622-s001.zip › Figures/Supplemental Figure.pptx]

## Slide 1
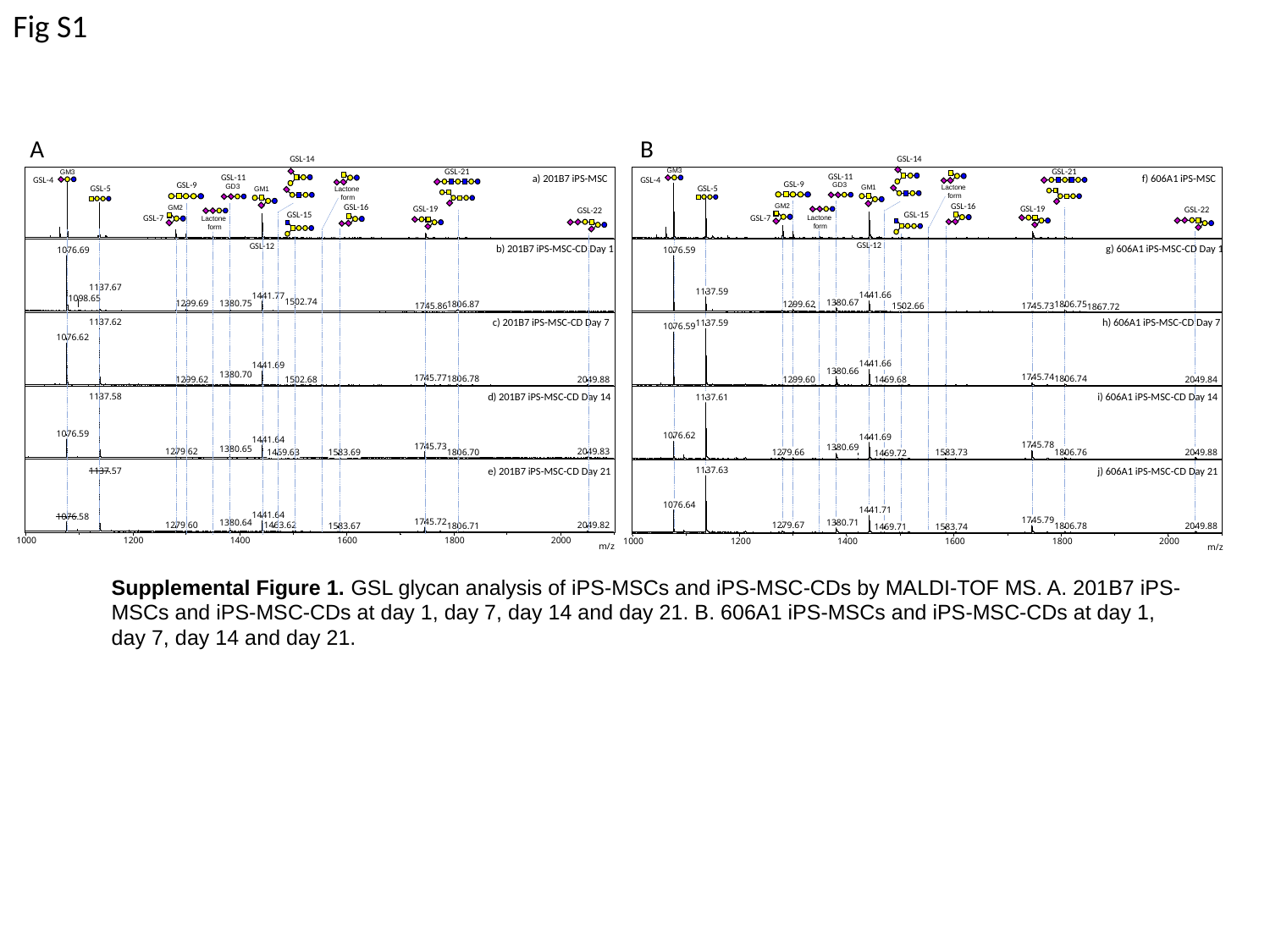

Fig S1
A
B
GSL-14
GSL-14
GSL-21
GM3
Lactone
 form
GD3
GM1
GM2
GSL-21
GM3
f) 606A1 iPS-MSC
a) 201B7 iPS-MSC
GSL-11
GSL-11
GSL-4
GSL-4
Lactone
 form
GSL-9
GSL-9
GD3
GSL-5
GSL-5
GM1
GSL-16
GSL-16
GSL-19
GSL-19
GM2
GSL-22
GSL-22
GSL-15
GSL-15
GSL-7
GSL-7
Lactone
 form
Lactone
 form
GSL-12
GSL-12
g) 606A1 iPS-MSC-CD Day 1
b) 201B7 iPS-MSC-CD Day 1
1076.59
1076.69
1137.67
1137.59
1441.66
1441.77
1098.65
1502.74
1380.67
1380.75
1299.69
1299.62
1806.87
1806.75
1745.86
1502.66
1745.73
1867.72
h) 606A1 iPS-MSC-CD Day 7
c) 201B7 iPS-MSC-CD Day 7
1137.62
1137.59
1076.59
1076.62
1441.66
1441.69
1380.66
1380.70
1745.74
1745.77
1806.74
1806.78
1299.62
1502.68
2049.88
1299.60
1469.68
2049.84
i) 606A1 iPS-MSC-CD Day 14
d) 201B7 iPS-MSC-CD Day 14
1137.58
1137.61
1076.59
1076.62
1441.69
1441.64
1745.78
1745.73
1380.69
1380.65
1279.62
2049.83
1279.66
1806.76
2049.88
1806.70
1583.73
1469.63
1583.69
1469.72
j) 606A1 iPS-MSC-CD Day 21
e) 201B7 iPS-MSC-CD Day 21
1137.63
1137.57
1076.64
1441.71
1441.64
1076.58
1745.79
1745.72
1380.64
1380.71
1279.60
1463.62
2049.82
1279.67
1583.67
1806.71
1806.78
2049.88
1469.71
1583.74
1000
1200
1400
1600
1800
2000
1000
1200
1400
1600
1800
2000
m/z
m/z
Supplemental Figure 1. GSL glycan analysis of iPS-MSCs and iPS‐MSC-CDs by MALDI-TOF MS. A. 201B7 iPS-MSCs and iPS‐MSC-CDs at day 1, day 7, day 14 and day 21. B. 606A1 iPS-MSCs and iPS‐MSC-CDs at day 1, day 7, day 14 and day 21.

## Slide 2
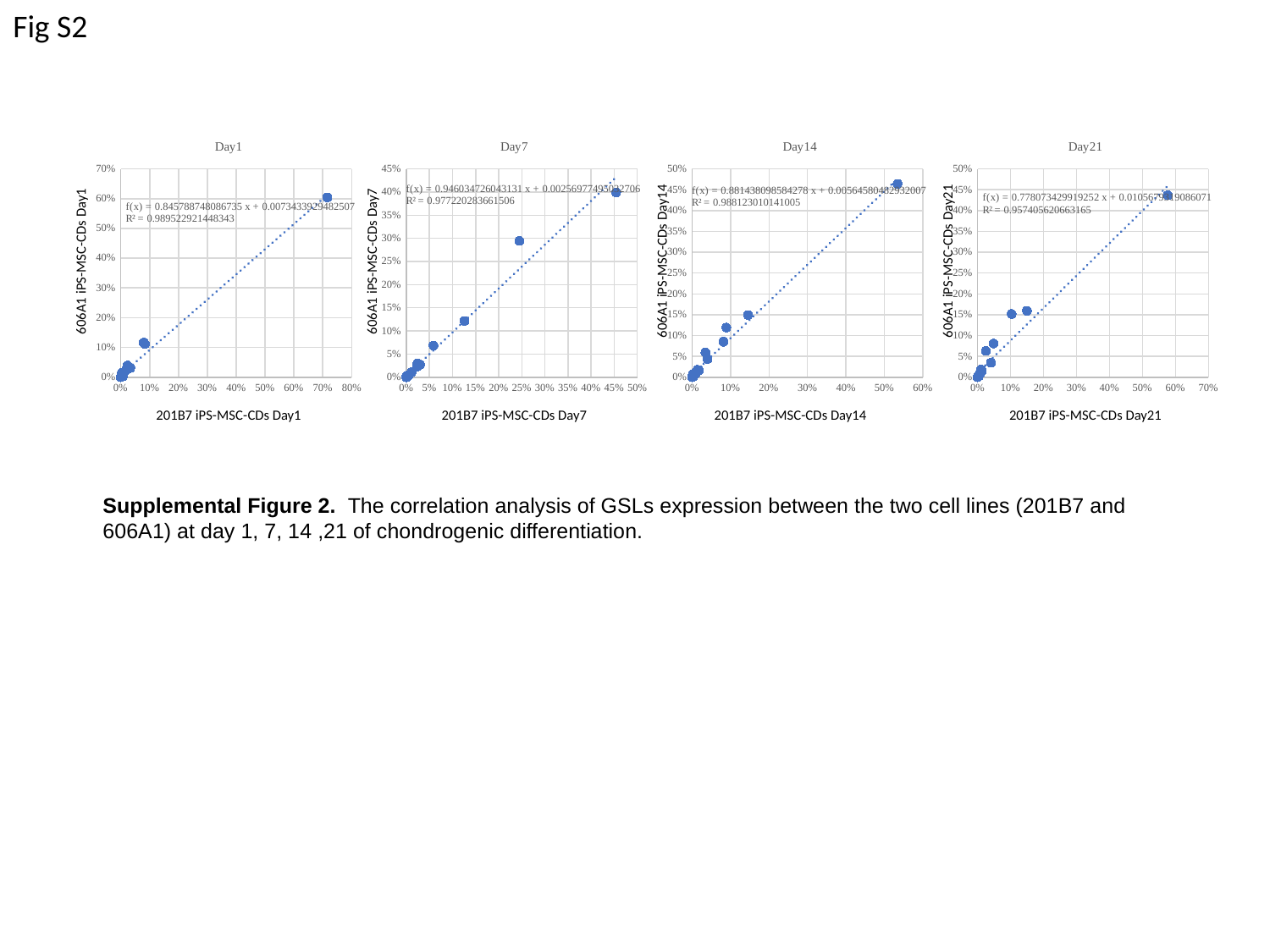

Fig S2
### Chart:
| Category | Day7 |
|---|---|
### Chart:
| Category | Day14 |
|---|---|
### Chart:
| Category | Day21 |
|---|---|
### Chart:
| Category | Day1 |
|---|---|606A1 iPS-MSC-CDs Day21
606A1 iPS-MSC-CDs Day1
606A1 iPS-MSC-CDs Day7
606A1 iPS-MSC-CDs Day14
201B7 iPS-MSC-CDs Day1
201B7 iPS-MSC-CDs Day7
201B7 iPS-MSC-CDs Day14
201B7 iPS-MSC-CDs Day21
Supplemental Figure 2. The correlation analysis of GSLs expression between the two cell lines (201B7 and 606A1) at day 1, 7, 14 ,21 of chondrogenic differentiation.

## Slide 3
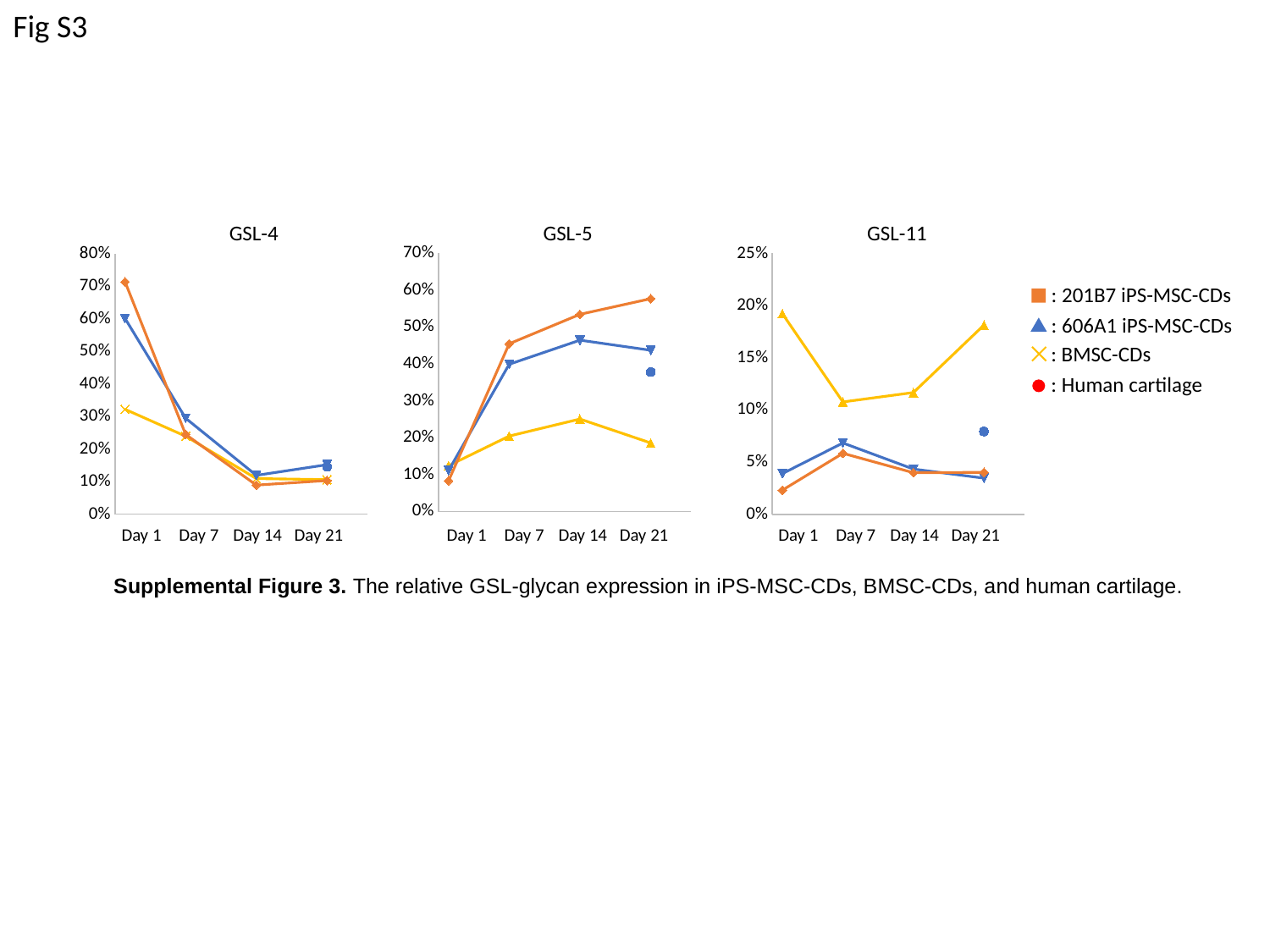

Fig S3
GSL-4
GSL-5
GSL-11
### Chart
| Category | | | | |
|---|---|---|---|---|
### Chart
| Category | | | | |
|---|---|---|---|---|
### Chart
| Category | | | | |
|---|---|---|---|---|: 201B7 iPS-MSC-CDs
: 606A1 iPS-MSC-CDs
: BMSC-CDs
: Human cartilage
Day 14
Day 7
Day 1
Day 21
Day 14
Day 7
Day 1
Day 21
Day 14
Day 7
Day 1
Day 21
Supplemental Figure 3. The relative GSL-glycan expression in iPS-MSC-CDs, BMSC-CDs, and human cartilage.
