# Supplementary material for: Alterations of Glycosphingolipid Glycans and Chondrogenic Markers during Differentiation of Human Induced Pluripotent Stem Cells into Chondrocytes
Source: Biomolecules. 2020 Dec 1;10(12):1622. doi: 10.3390/biom10121622 (PMC7760376; doi:10.3390/biom10121622)
Supplement: Supplementary file 1 [file biomolecules-10-01622-s001.zip › Figures/Figure ver 13.2.pptx]

## Slide 1
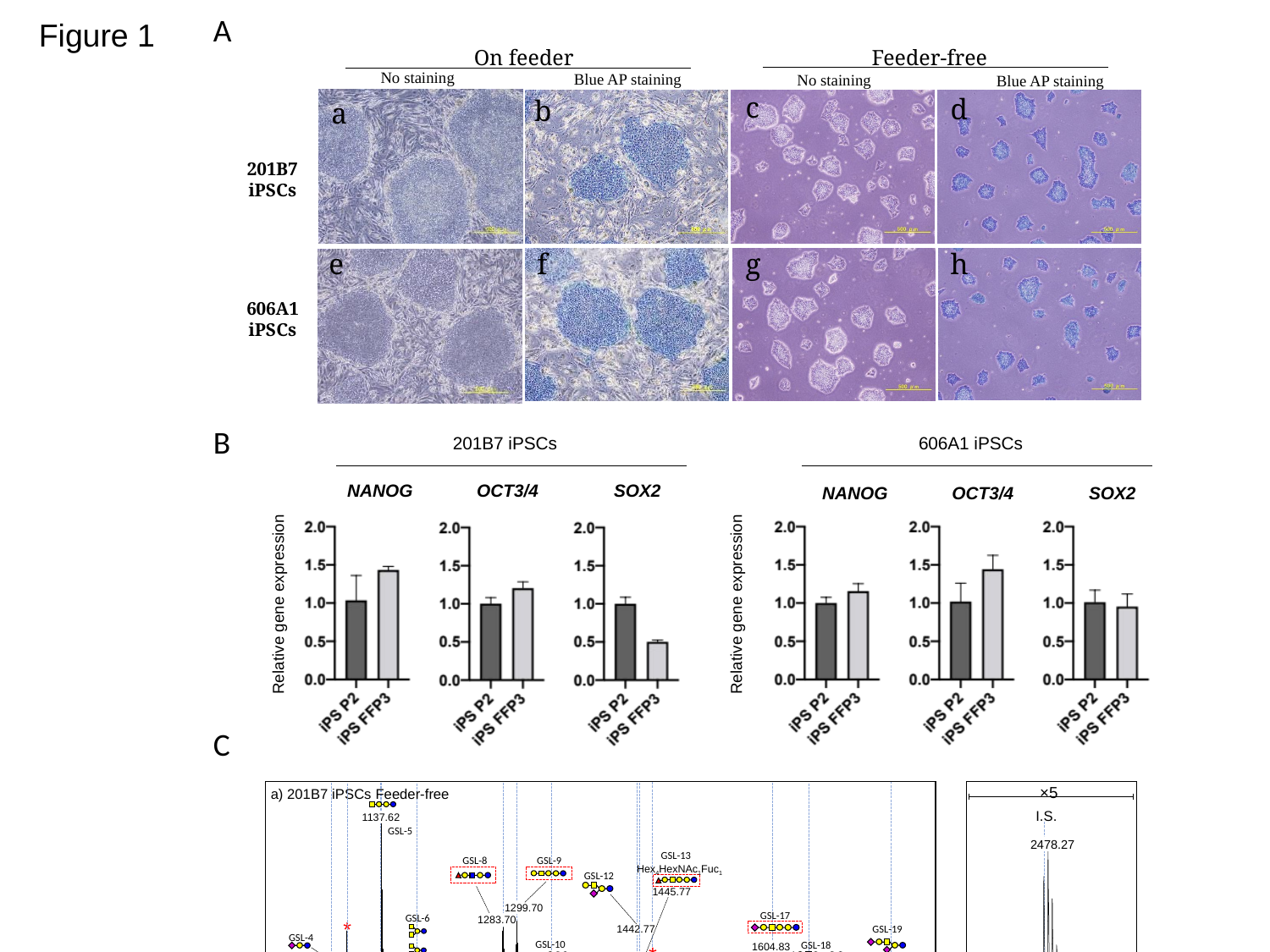

A
Figure 1
Feeder-free
On feeder
No staining
Blue AP staining
No staining
Blue AP staining
c
d
b
a
201B7
iPSCs
e
f
g
h
606A1
iPSCs
 B
201B7 iPSCs
606A1 iPSCs
NANOG
OCT3/4
SOX2
Relative gene expression
Relative gene expression
NANOG
OCT3/4
SOX2
 C
×5
a) 201B7 iPSCs Feeder-free
I.S.
1137.62
GSL-5
2478.27
GSL-13
GSL-9
GSL-8
Hex4HexNAc1Fuc1
GSL-12
1445.77
1299.70
GSL-17
GSL-6
*
1283.70
GSL-19
1442.77
GSL-4
GSL-10
GSL-18
*
1604.83
1178.65
1648.87
1077.57
1340.72
1747.90
b) 606A1 iPSCs Feeder-free
1138.05
1283.96
2478.60
1299.97
1446.05
1443.05
1605.13
1077.84
1583.07
1178.91
1649.16
1748.20
1341.00
m/z
2460
2470
2480
2490
1000
1100
1200
1300
1400
1500
1600
1700
m/z

## Slide 2
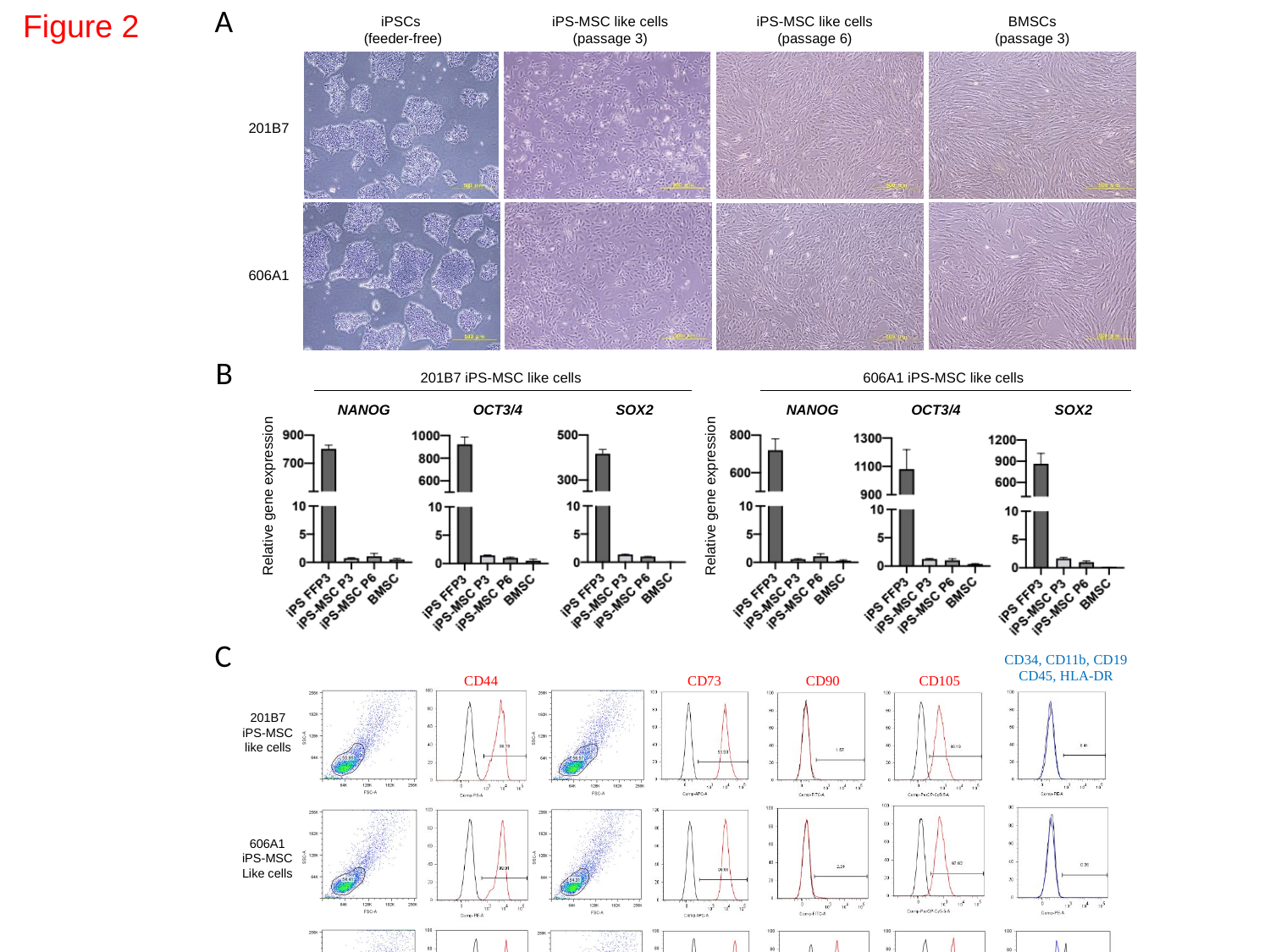

Figure 2
 A
iPSCs
 (feeder-free)
iPS-MSC like cells
(passage 3)
iPS-MSC like cells
(passage 6)
BMSCs
(passage 3)
201B7
606A1
 B
201B7 iPS-MSC like cells
606A1 iPS-MSC like cells
NANOG
OCT3/4
SOX2
NANOG
OCT3/4
SOX2
Relative gene expression
Relative gene expression
 C
CD34, CD11b, CD19
CD45, HLA-DR
CD44
CD73
CD90
CD105
201B7
iPS-MSC
like cells
606A1
iPS-MSC
Like cells
BMSCs

## Slide 3
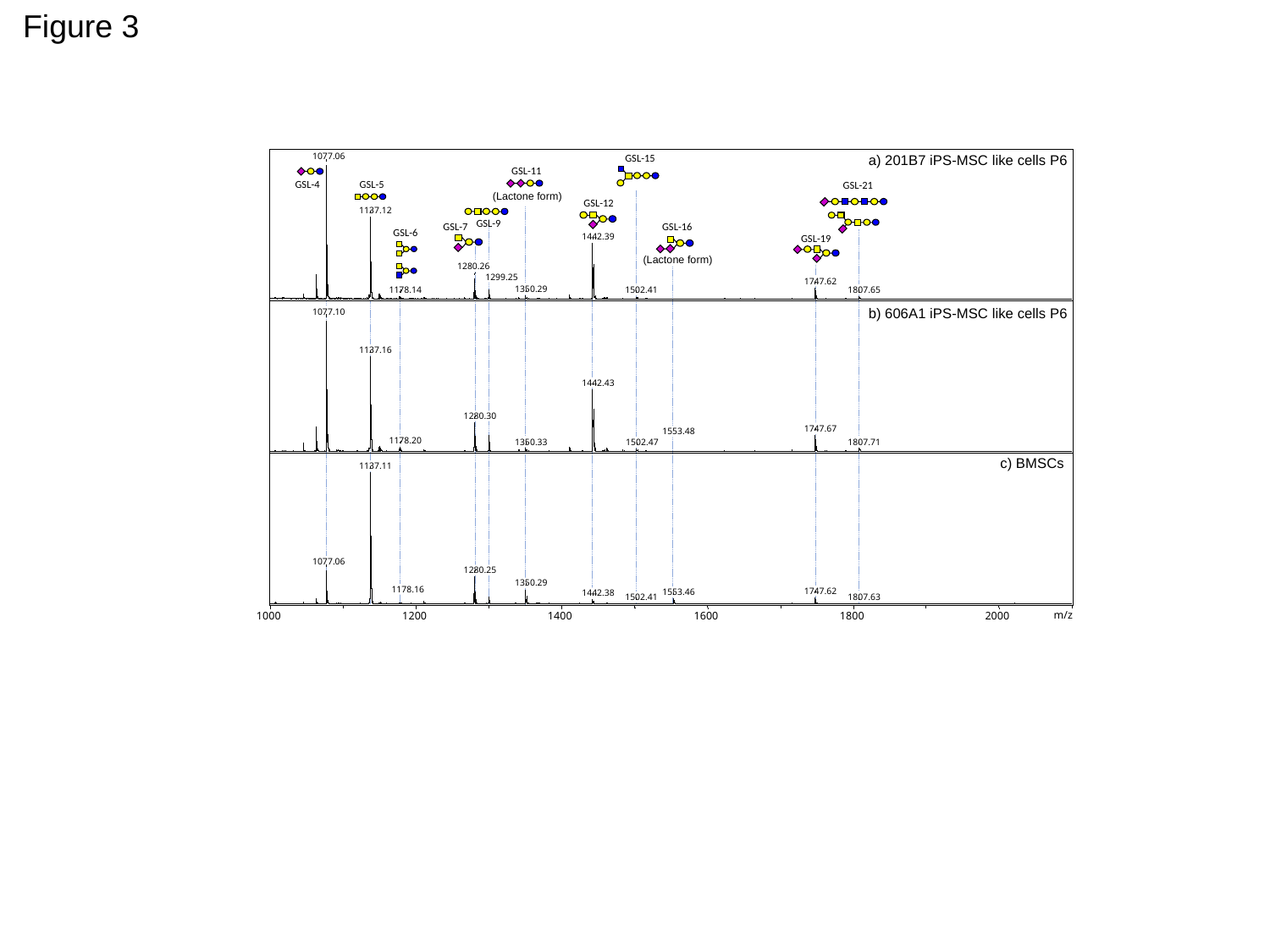

Figure 3
GSL-15
a) 201B7 iPS-MSC like cells P6
1077.06
GSL-11
GSL-5
GSL-4
GSL-21
(Lactone form)
GSL-12
1137.12
GSL-9
GSL-7
GSL-16
GSL-6
GSL-19
1442.39
(Lactone form)
1280.26
1299.25
1747.62
1350.29
1178.14
1807.65
1502.41
b) 606A1 iPS-MSC like cells P6
1077.10
1137.16
1442.43
1280.30
1747.67
1553.48
1178.20
1350.33
1807.71
1502.47
c) BMSCs
1137.11
1077.06
1280.25
1350.29
1178.16
1747.62
1553.46
1442.38
1502.41
1807.63
m/z
1000
1200
1400
1600
1800
2000

## Slide 4
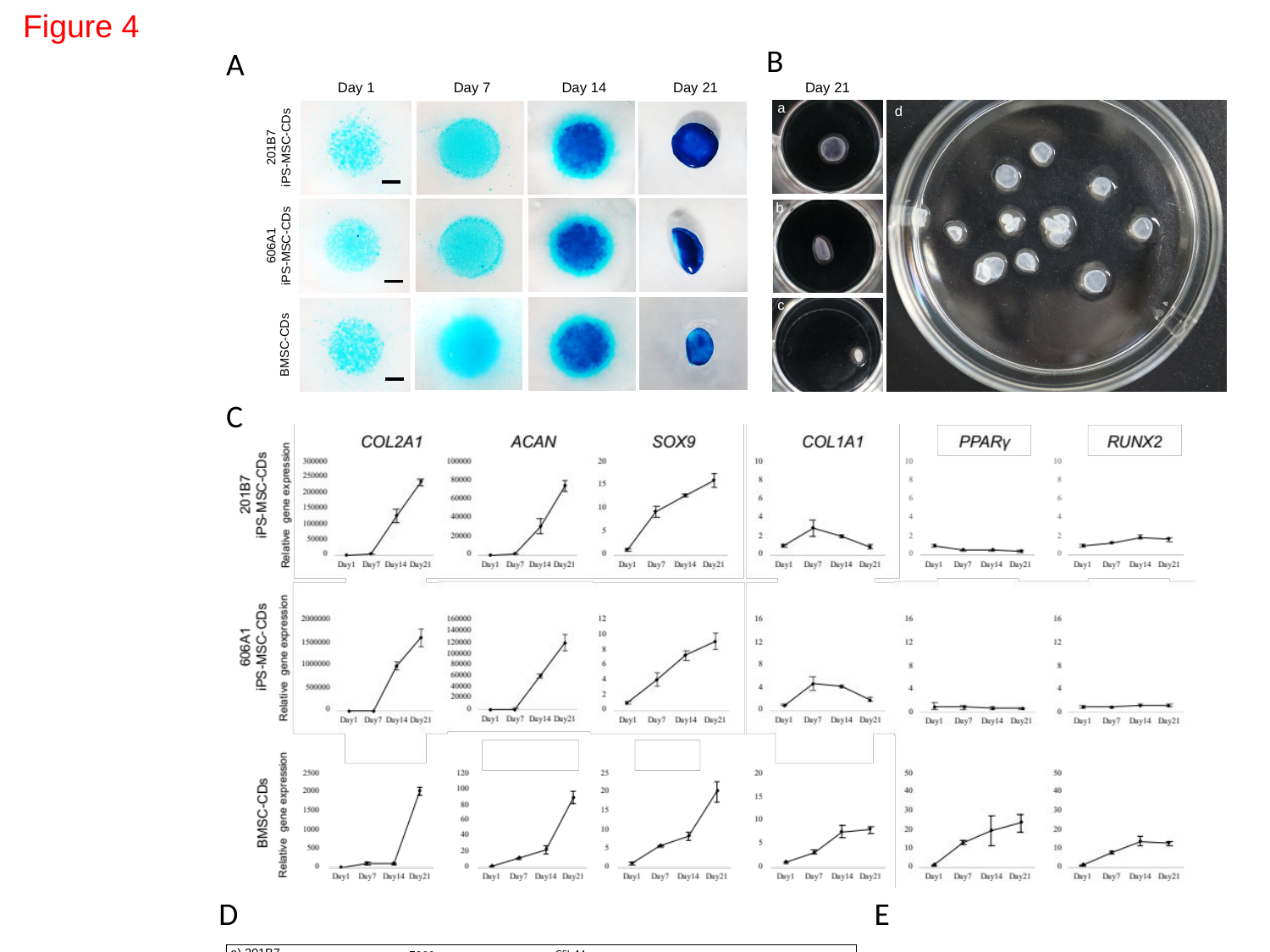

Figure 4
B
 A
Day 1
Day 7
Day 14
Day 21
Day 21
a
d
201B7
iPS-MSC-CDs
b
606A1
iPS-MSC-CDs
c
BMSC-CDs
 C
D
E
### Chart
| Category | GSL-1 | GSL-2 | GSL-3 | GSL-4 | GSL-5 | GSL-6 | GSL-7 | GSL-8 | GSL-9 | GSL-10 | GSL-11 | GSL-12 | GSL-13 | GSL-14 | GSL-15 | GSL-16 | GSL-17 | GSL-18 | GSL-19 | GSL-20 | GSL-21 | GSL-22 | GSL-23 |
|---|---|---|---|---|---|---|---|---|---|---|---|---|---|---|---|---|---|---|---|---|---|---|---|
| 201B7 iPS-MSC-CDs Day21 | 0.010469906491318473 | 0.025118878109978125 | 0.0040361454100302525 | 0.10305815048760057 | 0.5765648301930894 | 0.0 | 0.007902945942984442 | 0.0 | 0.003182519801130287 | 0.0 | 0.04034331701689187 | 0.14956580678292078 | 0.0 | 0.0020468563012500844 | 0.00031622748338747684 | 0.002965903299508417 | 0.0019327156700153078 | 0.0 | 0.048192343918088035 | 0.004618917690148653 | 0.0070840066607857155 | 0.012600528740872025 | 0.0 |
| 606A1 iPS-MSC-CDs Day21 | 0.018166407348596376 | 0.06296667953947124 | 0.004887848625217819 | 0.1516836546047816 | 0.43681429104658953 | 0.0 | 0.008587699021014524 | 0.0 | 0.0019471979330952143 | 0.000587440516655647 | 0.03480300267416957 | 0.15948490871025667 | 0.0 | 0.001751411431753996 | 0.0009969654762158604 | 0.00378017056554411 | 0.0016554138289914483 | 0.0 | 0.08073826086841449 | 0.005088192739012462 | 0.009803538918519636 | 0.015204175536951454 | 0.0010527406147481106 |
| BMSC-CDs Day 21 | 0.05655174498135155 | 0.14937780624596456 | 0.008816541227826963 | 0.10577996817478057 | 0.18577949426422863 | 0.0021692646732937434 | 0.01388826647123196 | 0.0 | 0.0034573930920026594 | 0.0010566897028861245 | 0.1813746035080784 | 0.07215584514873788 | 0.0 | 0.001486909978421519 | 0.0013413383632195726 | 0.04069862954415657 | 0.0028654766641709756 | 0.0 | 0.0856716555517424 | 0.0031668242147772324 | 0.004766343165540492 | 0.07959520502758845 | 0.0 |
| Normal Cartilage | 0.0776207390419623 | 0.11698874043492534 | 0.0 | 0.14517308442795337 | 0.37787372682898107 | 0.0 | 0.0 | 0.0 | 0.0 | 0.0 | 0.07946164287810219 | 0.09204711338194128 | 0.0 | 0.024793537737702067 | 0.018690607844299063 | 0.0 | 0.0 | 0.0 | 0.031795193040697535 | 0.0 | 0.02656766525924561 | 0.008987949124190282 | 0.0 |201B7
iPS-MSC-CDs
606A1
iPS-MSC-CDs
Human
cartilage
BMSC-CDs
a) 201B7
iPS-MSC-CDs Day 21
GSL-14
GSL-5
GSL-21
1137.66
GSL-3
GSL-11
GSL-12
GSL-4
GSL-2
GSL-22
GSL-19
GSL-7
GSL-16
1076.67
GSL-1
1441.74
934.62
1745.84
1380.75
772.57
1469.73
2049.96
975.63
1279.69
1806.83
1583.79
b) 606A1
iPS-MSC-CDs Day 21
1137.59
1076.60
1441.67
934.55
1745.75
1380.68
772.51
2049.86
975.56
1279.65
1806.76
1583.71
c) BMSC-CDs
Day 21
934.35
1137.42
1380.51
772.29
1441.51
1745.62
2049.72
1583.58
1279.47
1806.62
d) Human cartilage
1137.28
934.25
1076.28
1441.35
1380.36
1745.43
1806.43
1469.36
2049.49
800
1000
1200
1400
1600
1800
2000
m/z

## Slide 5
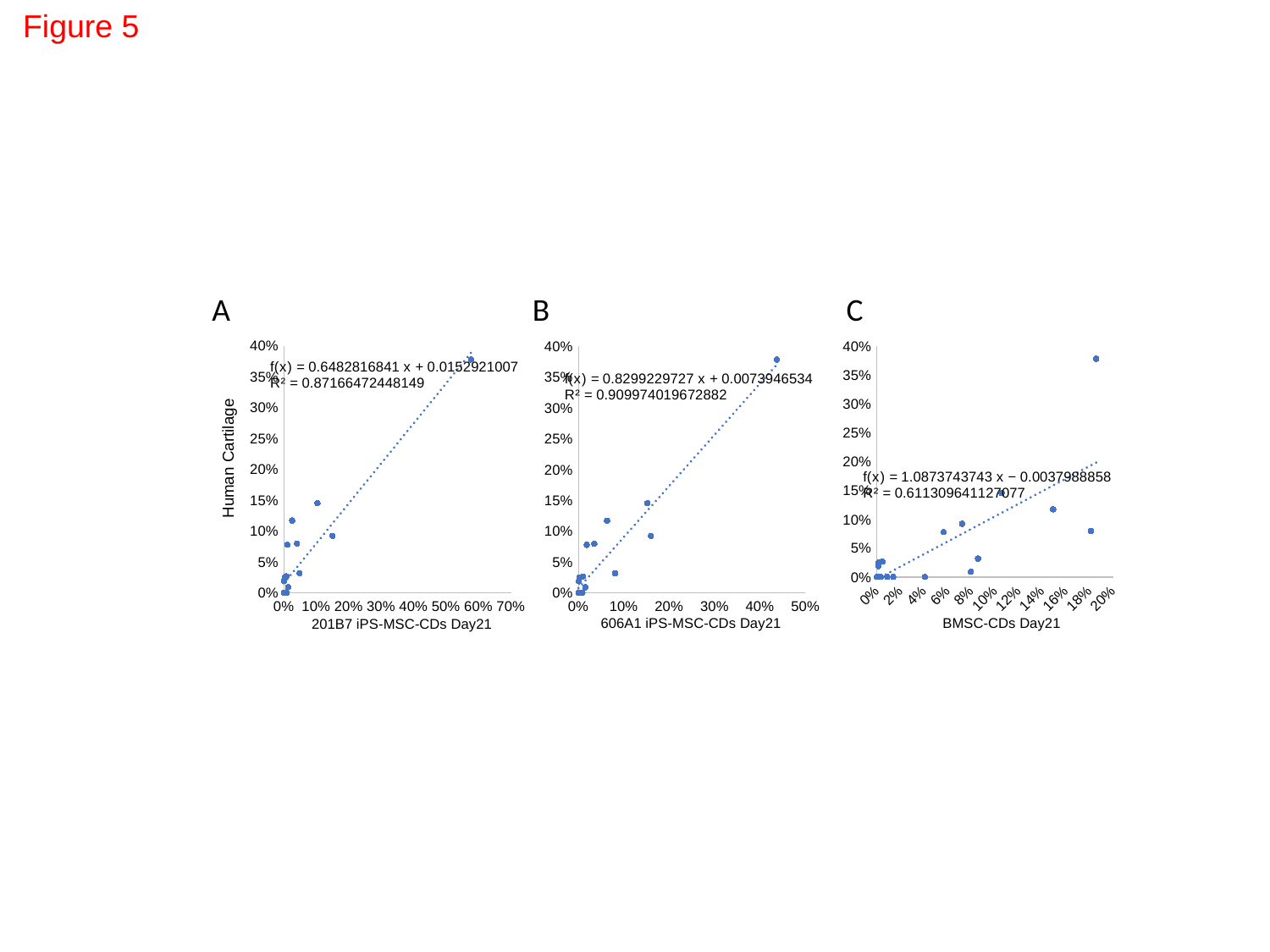

Figure 5
 A
 B
 C
### Chart
| Category | |
|---|---|
### Chart
| Category | |
|---|---|
### Chart
| Category | |
|---|---|Human Cartilage
606A1 iPS-MSC-CDs Day21
BMSC-CDs Day21
201B7 iPS-MSC-CDs Day21

## Slide 6
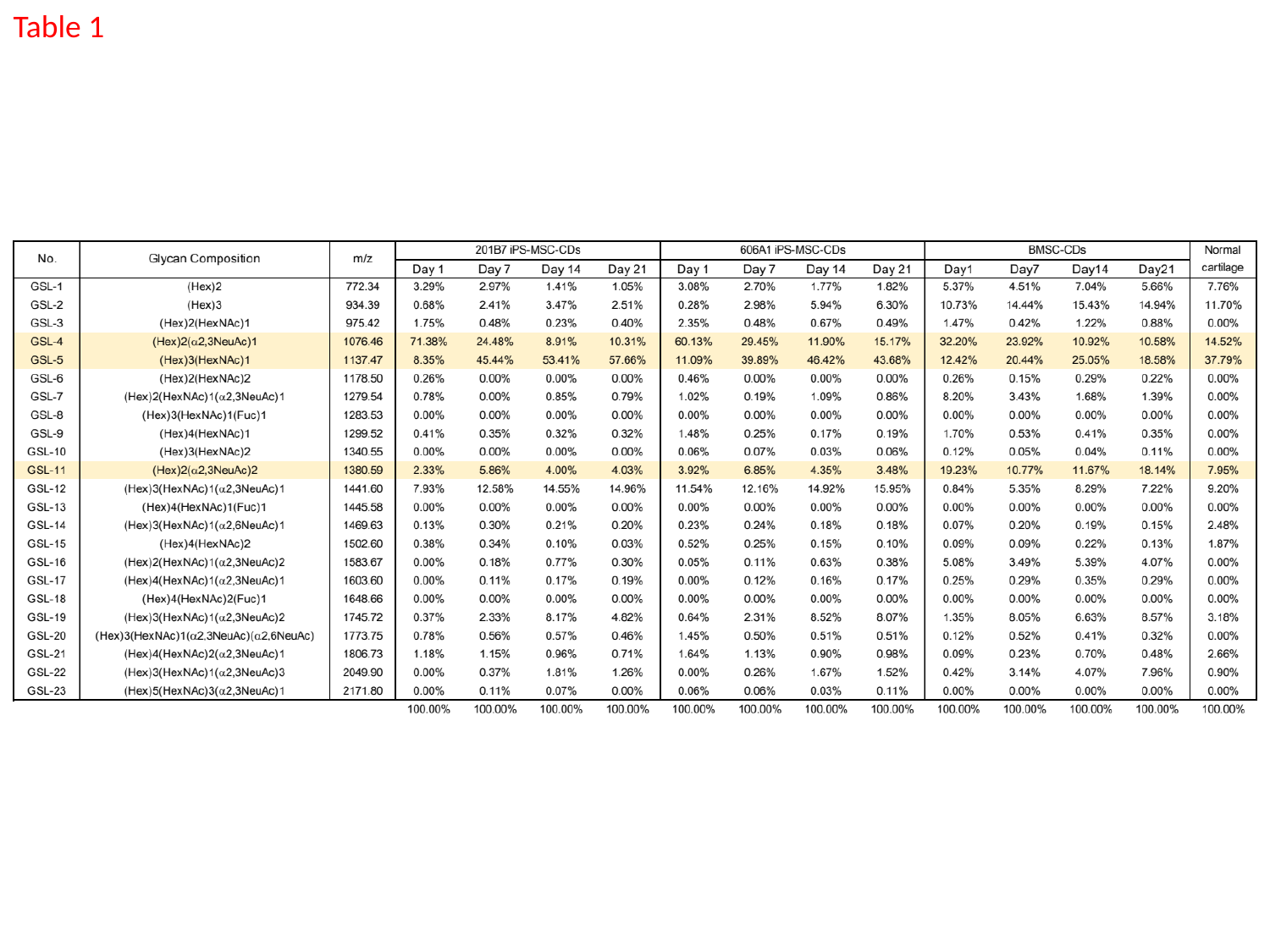

Table 1
